# Comparison of Chemical, Biological and Physical Quality Assessment of Indoor Swimming Pools in Shahrekord City, Iran in 2013

**DOI:** 10.5539/gjhs.v7n3p240

**Published:** 2014-11-30

**Authors:** Abdolmajid Fadaei, Masoud Amiri

**Affiliations:** 1 Department of Environmental Health Engineering, School of Health, Shahrekord University of Medical Sciences, Shahrekord, Iran; 2 Social Health Determinants Research Center, Shahrekrod University of Medical Sciences, Shahrekord, Iran

**Keywords:** microbiological indicators, hygiene, swimming pool, water quality

## Abstract

Previous studies have shown that mismanaged swimming pools could transmit water-borne diseases. The objective of the present study was the quality assessment of chemical, biological and physical characteristics of swimming pools in Shahrekord city, southwest of Iran.

The two main indoor swimming pools of Shahrekord city were considered during the summer and winter of 2013. The number of 459 samples were analysed from swimming pools, showers and dressing rooms for chemical, biological and physical quality assessment. The most prevalent fungi were *Aspergillus* (48.91%), *Penicillium* (22.9%), *Nocardia* (11.31%), *Cladosporium* (8.41%). *Rhizopus* (6.18%), *Scopulariopsis* (6.21%), *Fusarium* (5.31%), and *Mucor* (1.38%). The most fungal contamination sites for both swimming pools were showers.

Results showed that the values of total faecal coliform, *Pseudomonas aeruginosa, Legionalla, Escherichia coli* and Heterotrophic Plate Count (HPC) for both swimming pools exceeded the guidelines, except for *Staphylococcus aureus*. The correlation coefficient between bathers load and total faecal coliform, heterotrophic bacteria was 0.949. The turbidity, free residual chlorine, and hardness of both swimming pools were not compliance with standard guidelines. Therefore, the improvement of disinfection and cleaning procedures is necessary, due to the different users and daily bather loads of each pool, as well as monitoring the water quality and increasing of the knowledge of swimming pool users on the risks of these potential diseases.

## 1. Introduction

It is obvious that the use of swimming pools and similar recreational water environment has benefits for health and well-being; thus People would like to practice on water-related sport activities, and swimming pools and would go to sport, recreate, be relax and be socialized (Thomas & Murray 2008; Barna & Kadar 2012).

However, various studies have reported the chemical, biological, physical contaminations in swimming pools (Liguori et al., 2007; Le Cann, Bonvallot, Glorennec, Deguen, Goeury, & Le Bot, 2011; Abd El-Salam, 2012). There are many health risks related to the swimming pools such as the risk of drowning, trauma and injuries and risk of microbiological, chemicals and physical agents (WHO, 2006). Although, the most important related-risks of sports or recreational activities are derived from trauma and injuries, pathogenic microorganisms (faecal- and non- faecal origin) such as bacteria, protozoa, and viruses may also be presented in recreational waters. While microorganisms of non-faecal origin may include bacteria (*Pseudomonas*, *Staphylococcus aureus*, *Legionella*, *Mycobacterium* and *Leptospira*), Virus (*Molluscipoxvirus*, *Papillomavirus* and *adenoviruses*), Protozoa (*Naegleria fowleri, Acanthamoeba spp* and *Plasmodium*), Fungi (*Trichophyton* and *Epidermophyton floccosum*), faecal- origin microorganisms could also be entered the pool during cleaning process and be absorbed through skin or saliva, mucus including bacteria (*Shigella* and *E. coli 0157*), Virus (*Adenoviruses*, *Hepatitis A*, *Noroviruses* and *Enteroviruses*), Protozoa (*Giardia* and *Cryptosporidium*). In fact, they may be come from swimmers’ bodies or enter into the water when a person has an (accidental) faecal release contamination of water with such microorganisms originating from the skin, hair, saliva, urine or blood as potential causes of infection. Faecal pollution of the water is indeed the major microbiological risk of bathing in swimming pools, and recreational waters in general (WHO, 2006). Moreover, chemical risks are mainly those associated with the water-source origins like disinfection by products such as trihalomethanes, haloacetic acids, chlorate, nitrogen trichloride, precursors, bather origin (urine, sweat, dirt and lotions such as sunscreen, cosmetics, soap residues), management origin (disinfectants, pH correction chemicals and coagulants) (Zholdakova, Sinitsyna, Tulskaia, & Odintsov, 2007; Richardson et al., 2010; Vandyshev, 2010; Bonini, Bodina, Bonal, Bascucci, Pellino, & Castaldi, 2011; Keuten, Schets, Schijven, Verberk, & Van Dijk, 2012; Dallolio, Belletti, Agostini, Teggi, Bertelli, & Bergamini, 2013; Hansen, Zortea, Piketty, Vega, & Andersen, 2013; Pasquarella, 2013; Simard, Tardif, & Rodriguez, 2013). Reducing the amount of pollutants which may contaminate the pool water is possible, mainly through declining the disinfection made by products and chlorine. Generally, there are three main routes of exposure to the mentioned agents in swimming pools including ingestion, inhalation and dermal contact.

In addition, risk of physical agents such as feature pools and temperature is also important. Water temperatures ranging from 26 to 30 °C are the most comfortable temperature for most swimmers and upper or lower than this temperature interval might be dangerous for pregnant women and young children (WHO, 2006). Thus, microbiological parameters used for assessing pool water quality may include Faecal coliforms, *Staphylococcus aureus, Pseudomonas aeruginosa, Legionella*, *Escherichia* and *Heterotrophic Plate Count*. Staphylococci are common skin organisms which may be obtained from recreational waters (Valeriani, Giampaoli, Buggiotti, Gianfranceschi, & Romano Spica, 2012); it means that it is more likely to be found in pool waters, especially where the bathing load is heavy. It may also be considered as a risk indicator for skin, eye and ear diseases (Valeriani et al., 2012; Carmo et al., 2014). *Pseudomonads* and in particular *Pseudomonas aeruginosa* are able to grow in moist, warm conditions with low levels of organic nutrients. Therefore, they can be found in pool waters in different ways; i.e., directly by users or by cleaning methods, and then may become colonised with *Pseudomonas aeruginosa* (Valeriani, Giampaoli, & Romano Spica, 2014). Moreover, *Legionella pneumophila* have been found in whirlpool waters that have not been properly maintained (Napoli, Fasano, Iatta, Barbuti, Cuna, & Montagna, 2010). Everywhere which pool water quality is poor, *Escherichia coli* could be entered from swimmers bodies or an (accidental) faecal release (Keuten et al., 2012; Marion et al., 2010; Akhter, A., Imran, & Akhter, F., 2014; Zhan, Hu, & Zhu, 2014). In addition, Nanbkhsh and colleagues studied on the fungal contamination of four public indoor swimming pools in Iran and reported that the common identified fungi were *Candida* (22.9%), *Rhizopus* (4.16%) and *Aspergillus* (56.2%) in general, and in dressing rooms and bathrooms, *Alternaria*, *Cladosporium*, *Philophara* and *Trichophyton mentagrophytis* were isolated (Nanbkhsh, 2005). The objective of the present study was to investigate the chemical, biological and physical quality of swimming pools in Shahrekord city in southwest of Iran.

## 2. Method

### 2.1 Data Collection

A cross-sectional study was conducted in two indoor swimming pools of Shahrekord city in south west of Iran. 243 specimen were collected in the morning and the evening in the summer and the winter of 2013 from top, shallow and deep level for testing fungal and microbial by sterilized bottles and were immediately transferred to the lab. There were also 180 samples from showers, dressing rooms and bottom of the swimming pools for testing fungal, and 36 samples for testing chemical and physical water quality such as temperature, PH, chlorine residual, color, turbidity, dissolved oxygen, hardness, alkalinity, iron, manganese, nitrate, Magnesium, Sulphate, Calcium, Nitrite and Chloride. All samples were placed in cold storage (4–10 °C) immediately after sampling.

### 2.2 Physico-Chemical Analysis

Temperature, PH, chlorine residual, color, turbidity, dissolved oxygen were measured in situ. Alkalinity was determined by titration with sulfuric acid to a pH of 4.5. Hardness was determined by titration with a chelating agent, ethylenediaminetetraa- cetic acid (EDTA) and Eriochrome Black T as indicator, Calcium and Magnesium: The method allowed us to distinguish complexometry calcium magnesium samples by titration with disodium EDTA. The determination of chlorides is measured by the method of Mohr. Sulphate, Nitrate, Nitrite Diazotisation and Iron and Manganese were measured by Turbidity spectrophotometric method, Brucine sulphate spectrophotometric method, spectrophotometric method, Atomic absorption spectrophotometric method, respectively. The samples were examined based on standard methods (APHA, 2005). Both swimming pools had rectangular shape but with different size; i.e., length and width of Shahid Fazal swimming pool and Shohada swimming pool were 25 and 10, and 33 and 10 meters, respectively.

### 2.3 Microbiological Analysis

Heterotrophic bacteria count (HPC) were enumerated by pour plate technique and 1 ml of each sample was inoculated in Plate Count agar (APHA 2005). Incubation was performed at 37 °C for 24-42 hours and 22 °C for 5 days (APHA, 2005). *Faecal coliforms* per 100 ml at 44.5 °C (m-FC Agar) (Difco), *Staphylococcus aureus* per 100 ml at 36 °C (Chapman Agar) (Difco), *Staphylococcus* Selective Agar (Difco), *Pseudomonas aeruginosa* per 100 ml at 30 °C (*Pseudomonas* Selective Agar Base) (Difco). Cetrimide Agar. *Legionella* per 100 ml at 35 °C (Buffered Charcol Yeast Extract agar (BCYE) agar (Difco). *Escherichia coli* per 100 ml at 44.5 °C (EC-medium) (Difco). For all fungi, Corn meal agar and Sabouraud Destrose Agar with Chloramphenicol cultures were used (Merck). The plates were examined with intervals up to 7 days for assessing the growth situation. The results were statistically analyzed using chi-square test and correlation coefficients were also calculated.

## 3. Results

Table 1 shows the characteristics of swimming pool users by age and gender. The youngest age group was 6-9 years and the oldest age group was 18-39 years.

**Table 1:** Age distribution of interviewed swimming pool users

Age group(years)	Female(%)	Male(%)
6-9	8.29	4.5
10-13	10.2	10.8
14-17	18.9	20.6
18-39	40.1	4.1
40-45	13.8	14.6
>55	8.71	8.5

The results of physico-chemical and parameters were demonstrated in Table 2. The pH of the water were 8.08±0.29 and 7.85±0.24. The calcium of both swimming pools with a value of 186.83±26.04 and 163.66±25.83 mg/l. Other parameters such as temperature, alkalinity were conformed to limits established for water swimming pool standard are using in Iran (MHM, 2013).

**Table 2:** Mean and standard deviations values of physico-chemical parameters of studied pools

Parameter	Shahid Fazal pool	Shohada pool
Temperature (^0^C)	25.83±0.74	25.5±0.74
Turbidity (NTU)	1±0.15	1.85±0.25
Colour (TCU)	1.1±0.34	3.93±0.34
pH	8.08±0.29	7.85±0.24
Free residual chlorine (mg/L)	0.63±0.15	0.58±0.19
Iron (mg/L)	0.03±0.01	0.025±0.09
Manganese (mg/L)	0.15±0.05	0.2±0.057
Dissolved oxygen (mg/L)	8.38±0.69	7.28±0.78
Calcium (mg/L)	74.32±11.36	65.46±10.32
Magnesium (mg/L)	25.23±3.5	21.07±4.4
Sulphate (mg/L)	15.43±2.03	18.5±6.07
Chloride (mg/L)	64.65±6.33	70±4.56
Nitrate (mg/L)	12.75±3.13	13.26±2.14
Nitrite (mg/L)	0.005±0.001	0.006±0.001
Total alkalinity (mg/L CaCO_3_)	152.16±21.08	148.5±19.8
Methyl orange alkalinity (mg/L CaCO_3_)	152.16±21.08	148.5±19.8
Total hardness (mg/L CaCO_3_)	290.83±16.35	251±27.12
Calcium hardness (mg/L CaCO_3_)	186.83±26.04	163.66±25.83

NTU: Nephelomtric Turbidity Unit, TCU: True Color Unit.

Table 3 shows the results of microbiological analysis of swimming pools’ water. The microbiological indicators, total faecal coliform, *Pseudomonas aeruginosa, Staphylococcus aurreus*, *Legionalla*, and *Escherichia coli* per 100 ml of both swimming pools with a value of 7.5±3.4 and 14.4±2.1, 2.8±0.51 and 4.5±0.4, 1.5±0.44 and 3.1±0.8, 1.2±0.55 and 2.2±0.06, and 17.66±7.94 and 21.16±9.8, respectively. Other microbiological indicators was Heterotrophic Plate Count/1 ml 220±18.5 and 250±20.1.

**Table 3:** Microbiological analysis of swimming pool water in Shahrekord, Iran

Indicator	Iran guide lines(MHM)	WHOguidelines (MHRA)	European Union guidelines(EA)	Shahid Fazal pool	Shohada pool
Total faecal coliform/100 ml	<1	<1	0	7.5±3.4	14.4±2.1
Pesudomonad	<1	<1	0	2.8±0.51	4.5±0.4
aeruginosa/100 ml	<50	<50	Not foreseen	1.5±0.44	3.1±0.8
Staphylococcus aurreus/100 ml	<200	<200	0-100	220±18.5	250±20.1
Heterotrophic Plate	<1	<1	Not foreseen	1.2±0.55	2.2±0.06
Count/1 ml	Not foreseen	<1	0	17.66±7.94	21.16±9.8
Legionalla/100 ml					
Escherichia coli/100 ml					

Table 4 presents the results of fungal analysis of Shahid Fazal swimming pool, percentage of positive samples and number based on sample site. It shows that fungi could be present in Shahid Fazal swimming pool. The most prevalent fungi were *Aspergillus* (21.73%), *Penicillium* (7.24%), *Nocardia* (5.79%), *Fusarium* (5.31%), *Scopulariopsis* (4.83%), *Rhizopus* (2.89%), and *Cladosporium* (2.89%).The most contaminated locations to *Aspergillus* in Shahid Fazal swimming pool are shower (68.75%), dressing room (34.48%), bottom of the pool (20%), and water pool (5.51%). Most fungal species were isolated from showers, dressing rooms, bottom of the swimming pools, and water pool (21.25%, 14.49%, 5.31 and 11.59%, respectively).

**Table 4:** Distribution of fungal species isolated from Shahid Fazal swimming pool

Number of samples	Sample site	Aspergillus n(%)	Penicillium n(%)	Rhizopus n(%)	Scopulariopsis n(%)	Cladosporium n(%)	Nocardia n(%)	Fusarium n(%)
29	Dressing room	10(34.48)	10(4.48)	-	1(3.44)	2(6.89)	1(3.44)	6(0.68)

32	Shower	22(68.75)	3(9.37)	3(9.37)	3(9.37)	-	5(15.62)	3(9.37)

116	Water pool	7(5.51)	1(0.86)	3(9.37)	6(5.17)	1(0.86)	5(4.3)	1(0.86)

30	Bottom of the pool	6(20)	1(0.86)	-	-	3(10)	1(3.33)	1(3.33)

207	-	45(21.73)	15(7.24)	6(2.89)	10(4.83)	6(2.89)	12(5.79)	11(5.31)

n(%): number of positive samples, percentage of positive samples.

Table 5 demonstrates the results of fungal analysis of Shohada swimming pool, percentage of positive samples and number based on sample site. Fungi has also been shown to be present in Shohada swimming pool. The most prevalent fungi were *Aspergillus* (27.18%), *Penicillium* (15.66%), *Cladosporium* (5.52%), *Nocardia* (5.52%), *Rhizopus* (3.29%), *Candida albicans* (1.84%), *Scopulariopsis* (1.38%,) and *Mucor* (1.38%).The most contaminated sites to *Aspergillus* in Shohada swimming pool were shower (61.2%), dressing room (51.6%), bottom of the pool (42.85%), and water pool (9.44%); briefly, the higher fungal contamination of Shohada pool(66.82%) compared to Shahid Fazal swimming pool(50.72%).Most fungal species were isolated from showers, dressing rooms, bottom of the swimming pools, and water pool (21.25%, 21.19%, 12.9% and 11.52%, respectively).

**Table 5:** Distribution of fungal species isolated from Shohada swimming pool

Number of samples	Sample site	Aspergillus n(%)	Penicillium n(%)	Rhizopus n(%)	Scopulariopsis n(%)	Cladosporium n(%)	Nocardia n(%)	Mucor n(%)	Candida albicans n(%)
31	Dressing room	16(51.6)	13(41.93)	3(9.67)	-	6(9.35)	4(12.9)	-	4(12.9)

31	Shower	19(61.2)	8(25.8)	3(9.67)	4(12.9)	6(9.35)	4(12.9)	3(9.67)	-

127	Water pool	12(9.44)	1(0.86)	8(6.29)	-	-	4(14.3)	-	-

28	Bottom of the pool	12(42.85)	12(42.85)	4(14.28)	-	-	-	-	-

217	-	59(27.18)	34(15.66)	18(3.29)	3(1.38)	12(5.52)	12(5.52)	3(1.38)	4(1.84)

n(%): number of positive samples, percentage of positive samples.

## 4. Discussion

In this study, physico-chemical parameters were lower or higher than standard levels, so it is necessary to pay more attention to the physico-chemical parameters. There was no statistically significant difference between level of microbiological pollutants and seasons. Not taking a shower before swimming could increase the number of micro-organisms as well as sweat and chemicals and hair. While the turbidity and hardness of both swimming pools was higher than Iranian standard, the free residual chlorine of both swimming pools was less than Iranian standard.

Our results showed that the pool water pH was between 7.85 and 8.08. According to guidelines, the recommended pH for swimming pool water ranged from 7.2 to 8 (MHM, 2013). Low pH of water can indeed result in corrosive nature of water, skin and eye irritation, loss of chlorine, and skin stains in swimmers (Hoseinzadeh et al., 2013). The correlation coefficient between bathers load and total faecal coliform, heterotrophic bacteria was 0.949 (Casanovas-Massana & Blanch, 2013); thus, it has been advised to use it for investigation of hygienic conditions of swimming pools.

Taking a shower may reduce the number of micro-organisms as well as sweat and chemicals (like cosmetics), hair (which may transfer to the water).The turbidity of both swimming pools was more than Iranian standard (i.e., <0.5 NTU). In addition, the free residual chlorine of both swimming pools was less than standard of Iran (1–3 mg/l). The hardness of both swimming pools was, again more than standard of Iran (180–250 mg/l CaCO_3_). Other parameters such as, temperature, alkalinity were conformed to limits established for water swimming pool Iranian standard. The free residual chlorine < 3 mg/l and turbidity >0.5 NTU in the water pool were indicative of unsatisfactory management of the water disinfection and filtration process, because free residual chlorine may be unable to oxidize the organic compounds and kill the microorganisms that had enhanced the water while passing through the filters (Fadaei & Sadeghi, 2014). Free residual chlorine and turbidity were also desirable (reported by Nikaeen, Hatamzadeh, Vahid Dastjerdi, Hassanzadeh, Mosavi, & Rafie, 2010) and free available chlorine in ten indoor swimming pools in Taipei, Taiwan (0.3–0.7 mg/l) was lower than guidelines (Chu, Cheng, Wang, & Tsai, 2013). In our study, the main reason of low free chlorine residual were chlorination defect, presence ammonia nitrogen and organic matter. About 1 mg/l free chlorine residue is sufficient to reduce *Escherichia coli*, *Legionella pneumophila*, *Pseudomonas aeruginosa*, *Staphylococcus aureus*, and *Candida albicans* (Chu et al., 2013; Borgmann-Strahsen, 2003).

There was also a correlation between turbidity and bacteria load, different studies have related water quality and various microbiological indicators to variables such as bather load or physico-chemical status (Nikaeen et al., 2010; Ibarluzea, Moreno, Zigorraga, Castilla, Martinez, & Santamaria, 1998). Other physical–chemical parameters such as dissolved oxygen, alkalinity, and hardness have technical and economic effects on swimming pools.

The bacteriological quality of the pools was considered as acceptable or unacceptable according to the Iranian standards. The total number of faecal coliform, *P. aeruginosa*, *Legionalla*, *E. coli* and HPC for swimming pools were exceeded the standards, except for *Staphylococcus aureus* contamination (Nikaeen et al., 2010; Guida, Galle, Mattei, Anastasi, & Liguori, 2009; Amagliani, Schiavano, Stocchi, Bucci, & Brandi, 2013; Casanovas-Massana & Blanch, 2013). In another study, aerobic plate count, *Pseudomonas aeruginosa* and *Staphylococcus aureus* 32%, 15.5% and 10.2%, respectively were reported (Ibarluzea et al., 1998). In another study, most microbial species were isolated from swimming pools include: *P.aeruginosa*, *E. coli*, and *enterococci spp* (Casanovas-Massana & Blanch, 2013). Causes of microbiological contamination of swimming pools were irregular chlorination, deficiency in filtration, high load swimmers, low knowledge of users, and not taking a shower before entering the swimming pool. The results showed that the Shohada swimming pool was more contaminated than Shahid Fazal swimming pool. In this swimming pool, bather density would presumably be higher; thus, the contribution of faecal pollution and other pathogens by the bathers would also be higher (Casanovas-Massana & Blanch, 2013). In addition, the sanitary condition of the bathers would be more difficult to control. Consequently, the filtration and chlorination system could be insufficient to reduce microorganisms and faecal contamination. The most fungal contamination site for both swimming pools was showers. Most fungal species were isolated from swimming pools include *Aspergillus*, *Penicillium*, *Cladosporium*, *Nocardia*, *Rhizopus*, *Candida albicans*, *Scopulariopsis*, *Mucor*, and *Fusarium* in Shahrekord city, Iran, they were saprophyte fungi (Jafari, Ghaneian, Ehrampoush, & Zarei, 2013; Papadopoulou et al., 2013). *Candida* species was isolated from 1.84% of the water samples. In other countries such as Italy, Greece and South America, *Candida* species were also isolated from swimming pools (13.2%, 4.7%, 2.0%, respectively) (Papadopoulou et al., 2013). Similar fungal species (*C. albicans*, *Penicillium spp*., *Rhizopus spp*., *Aspergillus versicolor*, *Aspergillus niger*, *Fusarium spp*., *Trichophyton mentagrophytes*, and *Mucor spp*.,) have been reported from the water of swimming pools in two studies in Nigeria and Iran (Itah & Kpombok, 2004; Kazemi-Fard, Jandaghi, Safdari, & Azizi-Far, 2007). In Hamadan, a city of western Iran, it has been found that the most isolated fungi were *Cladosporium spp*., *Penicillium spp*., *Aspergillus spp*., *Alternaria spp*., *Aspergillus.niger* *spp*., *Rhodotorula spp* and *Phoma spp* (Hoseinzadeh et al., 2013). Many reports showed that absence of adequate disinfect in swimming pools water can lead to that water be considered as an important source of fungal diseases spreading. Presence of some infectious agents, saprophytic fungi and other microorganisms at floor or surfaces of many locations in swimming pools will lead to spread and transmit of certain diseases (Nanbakhsh et al., 2004; Jafari et al., 2013; Hoseinzadeh et al., 2013).

In conclusion, the prevention of risk in swimming pools needs a combination of approve pool design and construction, pool inspection and management, and public education; however, pools may present certain risks that must be taken into account for the safety of bathers and personnel. In recent years, much attention has focused on the risk of infection associated with contamination by faecal and non- faecal micro-organisms, to ensure a safer environment in these swimming pools. It is also essential to increase users’ knowledge and information of the risks in order to improve the correct behaviours. The monitoring and supervision of recreational water use is a principal step for surveillance and management of sufficient health conditions in swimming pools. It seems that the variability of the pool water quality is important and there is a necessity for continuous quality monitoring (Hoseinzadeh et al., 2013). Improvements in swimmers’ health will require changes in their knowledge, attitude and behaviour, thus, with social and behavioural science theories based interventions are important. The most common age group users of the swimming pools were 18-39 years, it is essential to conduct a public health education program for this age group. For example, taking a shower before entering a swimming pool is essential for maintenance of proper water and air quality and also to reduce the risk of biological and chemical contamination.
